# Safety, pharmacodynamics, and pharmacokinetics of multiple oral doses of delta-9-tetrahydrocannabinol in older persons with dementia

**DOI:** 10.1007/s00213-015-3889-y

**Published:** 2015-03-11

**Authors:** Amir I. A. Ahmed, Geke A. H. van den Elsen, Angela Colbers, Cornelis Kramers, David M. Burger, Marjolein A. van der Marck, Marcel G. M. Olde Rikkert

**Affiliations:** 1 Department of Psychogeriatric Medicine, Vincent van Gogh Institute, Overloonseweg 4, 5804 AV Venray, The Netherlands; 2 Department of Geriatric Medicine and Radboud Alzheimer Centre, Radboud University Medical Center, Nijmegen, The Netherlands; 3 Department of Pharmacology and Toxicology, Radboud University Medical Center, Nijmegen, The Netherlands; 4 Department of Pharmacy, Radboud University Medical Center, Nijmegen, The Netherlands; 5 Department of Internal Medicine, Radboud University Medical Center, Nijmegen, The Netherlands; 6 Department of Pharmacy, Canisius Wilhelmina Hospital, Nijmegen, The Netherlands

**Keywords:** Tetrahydrocannabinol (THC), Safety, Pharmacodynamics, Pharmacokinetics

## Abstract

**Rationale:**

Data on safety, pharmacodynamics, and pharmacokinetics of tetrahydrocannabinol (THC) are lacking in dementia patients.

**Methods:**

In this randomized, double-blind, placebo-controlled, crossover trial, we evaluated the safety, pharmacodynamics, and pharmacokinetics of THC in ten patients with dementia (mean age 77.3 ± 5.6). For 12 weeks, participants randomly received oral THC (weeks 1–6, 0.75 mg; weeks 7–12, 1.5 mg) or placebo twice daily for 3 days, separated by a 4-day washout period.

**Results:**

Only 6 of the 98 reported adverse events were related to THC. Visual analog scale (VAS) feeling high, VAS external perception, body sway-eyes-open, and diastolic blood pressure were not significantly different with THC. After the 0.75-mg dose, VAS internal perception (0.025 units; 95 % CI 0.010–0.040) and heart rate (2 beats/min; 95 % CI 0.4–3.8) increased significantly. Body sway-eyes-closed increased only after 1.5 mg (0.59°/s; 95 % CI 0.13–1.06). Systolic blood pressure changed significantly after both doses of THC (0.75 mg, −7 mmHg, 95 % CI −11.4, −3.0; 1.5 mg, 5 mmHg, 95 % CI 1.0–9.2). The median *T*_max_ was 1–2 h, with THC pharmacokinetics increasing linearly with increasing dose, with wide interindividual variability (CV% up to 140 %). The mean *C*_max_ (ng/mL) after the first dose (0–6 h) was 0.41 (0.18–0.90) for the 0.75-mg dose and 1.01 (0.53–1.92) for the 1.5-mg dose. After the second dose (6–24 h), the *C*_max_ was 0.50 (0.27–0.92) and 0.98 (0.46–2.06), respectively.

**Conclusions:**

THC was rapidly absorbed and had dose-linear pharmacokinetics with considerable interindividual variation. Pharmacodynamic effects, including adverse events, were minor. Further studies are warranted to evaluate the pharmacodynamics and efficacy of higher THC doses in older persons with dementia.

## Introduction

In recent years, there has been increased interest in the medical applications of delta-9-tetrahydrocannabinol (THC), the main psychoactive cannabinoid of the cannabis plant (*Cannabis sativa L.*). A number of studies have demonstrated its effectiveness in the management of clinical conditions that are very common in older people, such as neuropsychiatric symptoms (e.g., agitation and aggression) in dementia, pain (e.g., neuropathic and spasticity in multiple sclerosis), and anorexia (Frytak et al. 1979; Sallan et al. 1980; Campbell et al. 2001; Lynch and Campbell 2011).

These therapeutic effects of THC are mediated primarily by two cannabinoid receptors: CB1 and CB2 (Devane et al. 1988; Matsuda et al. 1990; Munro et al. 1993). CB1 receptors are mainly expressed in the basal ganglia, cerebellum, hippocampus, hypothalamus, and dorsal horn (Pertwee 2006), and CB2 receptors are primarily found on immune cells and tumor cells (Pertwee et al. 2010). THC also interacts with other receptors and neurotransmitters in the brain, such as acetylcholine, dopamine, serotonin, gamma-aminobutyric acid, glutamate, norepinephrine, prostaglandins, and opioid peptides (Baker et al. 2003). These broad and complex interactions underlie the potential pharmacological effects of THC as multitarget drug candidate for the management of behavior, mood, pain, and anorexia in patients with dementia. Oral, fixed-dose THC-based drugs have recently been developed. For example, dronabinol (Marinol®) and nabilone (Cesamet®) have been approved in North America and some European countries for appetite stimulation in AIDS-related anorexia, chemotherapy-induced nausea/vomiting, and pain. Namisol® is the most recently developed THC-based formulation in tablet form but has not yet gained marketing approval (Klumpers et al. 2012).

Unfortunately, preapproval clinical trials of oral THC excluded old persons from participation or did not include sufficient numbers, and most recent studies that included older participants did not perform separate analyses for the older subgroup (Ahmed et al. 2014b; van den Elsen et al. 2014, Dronabinol prescribing information 2014). Studies of the potential effectiveness of THC in older individuals should include assessment of its safety, and especially in individuals with dementia, many of whom are frail and vulnerable (Ahmed et al. 2014b). To date, only four small studies have investigated the safety and efficacy of THC as treatment for the neuropsychiatric symptoms of dementia (Volicer et al. 1997; Walther et al. 2006, 2011; Woodward et al. 2014). All studies found THC to be effective and safe in older people with dementia, but as the studies were either not randomized or included a limited number of patients, it is not possible to draw firm conclusions about the safe and effective use of THC in these individuals. Furthermore, none of the studies investigated the pharmacokinetics of THC in this population. We found only one study in the literature that evaluated plasma THC concentrations (peak levels only) in older individuals (age 51–78 years), but these individuals were not demented (Carroll et al. 2004). Drug pharmacokinetics and pharmacodynamics in older people may be altered by age-related physiological changes, multiple comorbidities, or use of other medications. Aging is accompanied by an increase in adipose tissue, a decrease in lean body mass, and a decrease in total body water (Corsonello et al. 2010), changes which increase the volume of distribution of lipophilic drugs such as THC. Moreover, a decrease in hepatic blood flow and the slower metabolism of older individuals can slow the elimination of lipophilic drugs, thereby potentially increasing exposure and side effects (Linnebur et al. 2005). In addition, dementia-related changes in brain volume, number of neurons, and alteration in neurotransmitter sensitivity make older patients with dementia more sensitive to drugs that act on the central nervous system (Corsonello et al. 2010). Taken together, we hypothesize that the administration of THC to older people with dementia may lead to a higher THC concentrations, which subsequently lead to an increase in pharmacodynamic effects, including adverse effects, compared with previously published data for young adults (Klumpers et al. 2012) or healthy older individuals without dementia (Ahmed et al. 2014a). Understanding the pharmacodynamics and pharmacokinetics of THC in older, frail, dementia patients will help clinicians to minimize side effects and maximize benefit. Therefore, the aim of the present study was to evaluate the safety, pharmacodynamics, and pharmacokinetics of multiple oral doses of THC in older persons with dementia.

## Methods

### Study design and participants

This study was part of a multicenter, phase II, repeated crossover, randomized, double-blind, placebo-controlled, multiple-dose escalation trial of the effectiveness of THC in the treatment of the neuropsychiatric symptoms of dementia [http://www.clinicaltrials.gov, clinical trial identifier number NCT01302340]. The study was carried out at the Radboud University Medical Center, the Netherlands. Results concerning the effectiveness of THC in the management of the neuropsychiatric symptoms of dementia will be reported separately.

**Fig. 1:**
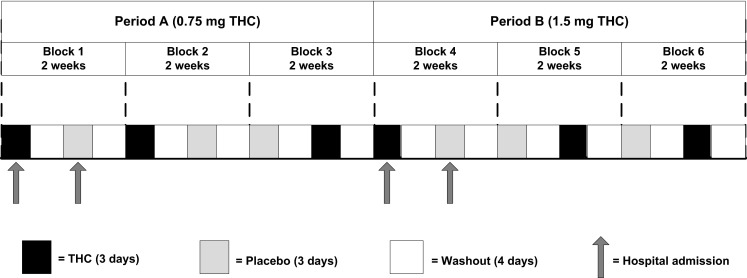
Overview of the treatment period (THC and placebo were administered at random) (this is an example of random allocation of treatment)

Figure 1 provides an overview of the study design. The study consisted of two treatment periods, A and B. Each period consisted of three treatment blocks, resulting in a total of six blocks (period A, blocks 1 to 3; period B, blocks 4 to 6). Each block lasted 2 weeks, giving a total study duration of 12 weeks. In each block, participants received oral Namisol®, a novel THC in tablet form (Klumpers et al. 2012), and matching placebo (ratio 1:1) in a double-blind crossover manner for 3 days, separated by a 4-day washout period. In period A, patients received 0.75 mg THC twice daily, and in period B, the dose was increased to 1.5 mg twice daily. Namisol® and placebo were identical in appearance and taste, and both were taken under nonfasting conditions with water at 10 a.m. and 4 p.m. Study participants stayed overnight at the study site on the three intervention days (THC and placebo) of blocks 1 and 4 for safety reasons and to facilitate blood sampling, resulting in a total of four 3-day admissions. The randomization codes were generated by an independent pharmacist, using a computer algorithm for random numbers. Sponsor, investigators, study staff, and participants were masked to assignment.

Participants had been diagnosed with dementia type Alzheimer, vascular dementia, or mixed Alzheimer/vascular dementia, according to the National Institute of Neurological and Communicative Disorders and Stroke–Alzheimer’s Disease and Related Disorders Association (NINCDS-ADRA) (McKhann et al. 2011) or Association Internationale pour la Recherché et l’Enseignement en Neurosciences (NINCDS-AIREN) criteria (Román et al. 1993). All patients had had clinically relevant neuropsychiatric symptoms, including at least agitation and/or aggression, in the past 30 days (Neuropsychiatric Inventory score ≥10) (Cummings et al. 1994), and had an informal caregiver who looked after the participant at least once a week. Main exclusion criteria were major psychiatric disorders (e.g., major depression or suicidal ideation, psychosis, mania, or current delirium), current history of severe comorbidities, frequent falling due to orthostatic hypotension, history of current alcohol or drug abuse, and use of tricyclic antidepressants, opioids, or drugs from a predesigned list of cytochrome (CYP)2C9, CYP2C19, and CYP3A4 inhibitors. Written informed consent was obtained from participants (if they were able to consent and to sign) and their legal representatives. The study was approved by the local ethics committee and was performed according to the International Conference on Harmonization guideline for good clinical practice, the ethical principles of the Declaration of Helsinki, and relevant Dutch laws and regulations.

### Safety and tolerability assessments

The safety and tolerability of THC were assessed subjectively and objectively, by evaluating the incidence and severity of adverse events, carrying out physical examinations, laboratory tests (hematology and clinical chemistry), and a 12-lead electrocardiogram, and assessing vital signs. The psychedelic effects were assessed with visual analogue scales (VAS), and body sway (postural stability) was measured using the SwayStar™ (see details below). During the study period, adverse events reported by patients and caregivers or observed clinically were recorded with regard to their time of onset, severity, duration, and causal relationship to study drugs. The causality was assessed by a research physician, blinded to treatment allocation, using a five-point scale: (1) unrelated, adverse event was clearly not related to the intervention; (2) unlikely, adverse event was doubtfully related to the intervention; (3) possible, adverse event may be related to the intervention; (4) probable, adverse event was likely related to the intervention; and (5) definite, adverse event was clearly related to the intervention. A serious adverse event was defined as any event that was fatal or life-threatening, that required (prolonged) hospitalization, or that resulted in persistent or significant disability or incapacity. All adverse events were coded using the Medical Dictionary for Regulatory Activities.

### Pharmacodynamic effects

The scores for psychedelic effects, body sway, and vital signs were used to evaluate the pharmacodynamic effects of THC.

1. Psychedelic effects: The Bowdle VAS for psychedelic effects was used to evaluate feeling high, internal perception (inner feelings that do not correspond with reality, including mistrustful feelings), and external perception (misperception of an external stimulus or change in awareness of surroundings) (Bowdle et al. 1998; Zuurman et al. 2008). Subjects were asked to score their perceptions on a 100-mm horizontal line, with “0” indicating no effect and “100” indicating extreme effect. The VAS was assessed 1 and 3 h after dosing on day 1 of weeks 1, 2, 7, and 8, in patients who were able to understand the instructions and perform the task. A recent study showed that individuals with dementia can use the VAS in a similar way to those without dementia (Arons et al. 2013).
2. Body sway: Body sway was assessed within 2 h of dosing on the second day of admission of weeks 1, 2, 7, and 8. Body sway was measured (30 s eyes open and 30 s eyes closed) with the SwayStar™, a wireless device attached to the trunk (http://www.b2i.info/web/index.htm).
3. Vital signs: Systolic and diastolic blood pressure and heart rate were measured on day 1 of weeks 1, 2, 7, and 8, before and at 15, 30, 45 min, and 1, 2, 3, and 4 h after the first dose.

### Blood sampling and laboratory analysis

Venous blood samples were collected during hospital admission before and at 11, 30, 45 min, and 1, 1.5, 2, 3, 4, and 6 h after the first dose, and before and at 11, 30, 45 min, and 1, 1.5, 2, 3, 4, 6, and 18 h after the second dose (in total covering a 24-h period). Plasma was separated by centrifugation (2000 × *g*, 4 °C, 10 min) and stored at −80 °C until analysis. After unblinding, blood samples collected in the THC treatment period were analyzed at the Analytisch Biochemisch Laboratorium b.v. (Assen, the Netherlands), using liquid chromatography with tandem-mass spectrometer detection. The lower limit of quantification was 0.1 ng/mL for THC and its active metabolite 11-OH-THC. The analysis was performed using a validated assay according to good laboratory practice standards (Guidance for Industry: Bioanalytical Method Validation 2001; Viswanathan et al. 2007).

### Pharmacokinetic analysis

Noncompartmental analysis was performed using Phoenix WinNonlin software version 6.3 (Certara, L.P./Pharsight Ltd) to determine the pharmacokinetics of THC and 11-OH-THC. The following pharmacokinetic parameters were calculated for the 24-h period: terminal half-life (*t*_1/2_), area under the curve (AUC) from 0 to 24 h (AUC_0–24 h_), and apparent clearance (CL/F, being the dose/AUC_0–24 h_). The following parameters were calculated for the two curves (curve 1, 0–6 h after the first THC dose; curve 2, 6–24 h after the second dose) separately: the maximum plasma concentration (*C*_max_), the time to reach *C*_max_ (*T*_max_), AUC from 0 to 6 h (AUC_0–6 h_), and AUC from 6 to 24 h (AUC_6–24 h_), using the linear-up log-down trapezoidal rule. Concentration-time graphs were plotted for the two doses. Geometric means plus 95 % confidence intervals were calculated for each pharmacokinetic parameter for each dose. The coefficients of variation (CV%) of the geometric means were calculated to describe the interindividual variability in pharmacokinetic parameters. The geometric mean ratio (GMR) plus 90 % confidence intervals of AUC_0–24 h_, CL/F, and *t*_1/2_ of the 1.5-mg dose versus the 0.75-mg dose were also calculated.

### Statistical analysis

This study is descriptive and explorative, and therefore, no sample size calculation was performed. Descriptive statistics were used to describe the study population. Continuous data are expressed as means ± standard deviation (±SD), and categorical data are expressed as frequencies and percentages. The compliance to study medication was calculated for the whole study sample. Differences in adverse event rates between THC and placebo were compared by Wilcoxon signed ranks test. The VAS scores were clustered and log-transformed, and the scores are expressed as units, as described previously (Zuurman et al. 2008; Klumpers et al. 2012). The 90 % range of pitch velocity (anterior–posterior movements) scores of the SwayStar™ was used to analyze body sway. Scores are given in degrees per second. The VAS, body sway, and vital signs scores were analyzed in relation to the THC dose, using linear mixed models with participants as a random effect. Statistical analyses were performed using SAS™ software, version 9.2 (SAS Institute, Inc., Cary, NC, USA).

## Results

### Participants

The data of ten patients with dementia were analyzed. Their demographic characteristics are summarized in Table 1. The mean age of participants was 77.3 ± 5.6 years; their mean body mass index was 25.7 ± 2.7 kg/m^2^; seven participants were men; and nine participants had Alzheimer’s disease. Overall, treatment compliance to study medication was high, and almost 98 % (THC 99 %; placebo 97.5 %) of the trial drugs were taken.

**Table 1:** Baseline demographic characteristics

Characteristics	*n* = 10
Male, *n* (%)	7 (70)
Age, mean (SD) (years)	77.3 (5.6)
BMI, mean (SD) (kg/m^2^)	25.7 (2.7)
Ethnicity, *n*	
Caucasian	9
Other	1
Type of dementia, *n*	
Alzheimer	9
Vascular	0
Mixed	1
MMSE score, mean (SD)	18.5 (6.0)
Smokers, *n*	0
Comorbidities, *n*	
Cardiac rhythm disorder	5
Hypertension	5
Ventricular hypertrophy	3
Diabetes	2
Electrolyte disturbances	2
Kidney function disorder	2
Vitamins deficiency	2
Hypercholesterolemia	1
Liver function disorder	1
Orthostatic hypotension	1
Medications, *n*	
Antidementia drugs^a^	16
Memantine	9
Rivastigmine	5
Galantamine	2
Antihypertensives^a^	11
Anticoagulants	4
Blood glucose lowering drugs	3
Antidepressants	1
Antiepileptics	1
Antipsychotics	1
Proton pump inhibitor	1
Other	12

^a^Some participants used a combination of drugs within the same medication group

### Safety and tolerability assessments

All participants completed the study as scheduled. In general, THC was safe and well tolerated by these older individuals with dementia. In total, 98 adverse events were reported during the study period. More adverse events were reported with placebo (55 adverse events) than with THC (43 adverse events) (period A, 0.75 mg THC 21 adverse events and placebo 30 adverse events, *P* = 0.290; period B, 1.5 mg THC 22 adverse events and placebo 25 adverse events, *P* = 0.435).

Thirteen (13 %) of the reported adverse events were considered to be possibly (*n* = 12) or probably (*n* = 1) related to study drugs (THC and placebo). Of these, only six adverse events (6 % of total adverse events) were considered to be (possibly) related to THC, two with 0.75 mg (dizziness and fatigue in one patient each), and four with 1.5 mg (agitation in three patients and fatigue in one patient). All were mild and transitory in nature. There were no THC-related serious adverse events. THC treatment was not associated with changes in the patients’ physical state, laboratory test results (hematology and clinical chemistry), or ECG parameters (e.g., QT and RR intervals).

### Pharmacodynamic results

THC did not cause significant changes in scores for VAS feeling high, VAS external perception, body sway with eyes open, and diastolic blood pressure (Table 2). The 0.75-mg dose, but not the 1.5-mg dose, was associated with a statistically significant increase in VAS internal perception scores (0.025 units, 95 % CI 0.010, 0.040). The 1.5-mg dose, but not the 0.75-mg dose, significantly increased body sway with eyes closed (0.59°/s, 95 % CI 0.13, 1.06). The 0.75-mg dose significantly decreased systolic blood pressure (−7.2 mmHg, 95 % CI −11.4, −3.0), whereas the 1.5-mg dose significantly increased systolic blood pressure (5.1 mmHg, 95 % CI 1.0, 9.2). Heart rate increased significantly after the administration of the 0.75-mg dose only (2 beats/min, 95 % CI 0.4, 3.8). None of the changes in the pharmacodynamic parameters was associated with an adverse event.

**Table 2:** Pharmacodynamic effects of THC doses

Parameters^a^	THC 0.75 mg versus placebo (*n* = 10)	THC 1.5 mg versus placebo (*n* = 10)
VAS feeling high (U)^b^	−0.010 [95 % CI −0.037; 0.017]; *P* = 0.47	0.002 [95 % CI −0.024; 0.028]; *P* = 0.90
VAS external perception (U)^b^	0.012 [95 % CI −0.005; 0.029]; *P* = 0.16	−0.014 [95 % CI −0.031; 0.003]; *P* = 0.11
VAS internal perception (U)^b^	0.025 [95 % CI 0.010; 0.040]; *P* = 0.001^c^	−0.002 [95 % CI −0.014; 0.010]; *P* = 0.75
Body sway, eyes open (°/s)	0.37 [95 % CI −1.31; +2.10]; *P* = 0.63	0.26 [95 % CI −0.91; 1.44]; *P* = 0.67
Body sway, eyes closed (°/s)	0.61 [95 % CI −0.63; +1.85]; *P* = 0.30	0.59 [95 % CI +0.13; +1.06]; *P* < 0.05^c^
Systolic blood pressure (mmHg)	−7.2 [95 % CI −11.4; -3.0]; *P* < 0.001^c^	5.1 [95 % CI 1.0; 9.2]; *P* < 0.05^c^
Diastolic blood pressure (mmHg)	0.2 [95 % CI −2.0; 2.3]; *P* = 0.86	−0.1 [95 % CI −2.2; 2.0]; *P* = 0.92
Heart rate (beats/min)	2.1 [95 % CI 0.4; 3.8]; *P* < 0.05^c^	−0.4 [95 % CI −2.0; 1.3]; *P* = 0.66

^a^All parameters are presented as mean [95 % confidence intervals (CI)]; *P* values

^b^Log-transformed visual analog scale (VAS) [scores in mm + 2]. Scores are given in units (U)

^c^Statistically significant *P* values (α = 0.05)

### Pharmacokinetic results

Pharmacokinetic parameters are summarized in Tables 3 and 4. The data of one person were excluded because no blood samples were taken after the first THC dose of 0.75 mg, and only a limited amount of blood was taken after the second dose. Although one subject was non-Caucasian, his pharmacokinetic data were within the range of the others.

**Table 3:** Pharmacokinetic parameters of THC and 11-OH-THC

Parameters^a^	THC		11-OH-THC	
	0.75 mg (*n* = 9)	1.5 mg (*n* = 10)	0.75 mg (*n* = 9)	1.5 mg (*n* = 10)
AUC_0–24_ (ng h/mL)	2.21 [96] (1.19–4.12)	4.66 [122] (2.35–9.25)	3.86 [46] (2.76–5.42)	8.92 [50] (6.35–12.54)
CL/F (L/h)	0.68 [96] (0.36–1.26)	0.64 [122] (0.32–1.28)	0.39 [46] (0.28–0.54)	0.34 [50] (0.24–0.47)
*t*_1/2_ (h)	5.08 [39] (3.81–6.77)	5.06 [37] (3.92–6.54)	7.80 [31] (6.19–9.82)	6.77 [61] (4.54–10.10)
*C*_max_ curve 1 (ng/mL)	0.41 [138] (0.18–0.90)	1.01 [112] (0.53–1.92)	0.56 [62] (0.36–0.87)	1.21 [61] (0.90–1.64)
*T*_max_ curve 1 (h)	1.5 (0.75–3.08)	1.01 (0.5–2.2)	1.5 (0.75–3.08)	1.76 (0.75–3.02)
*C*_max_ curve 2 (ng/mL)	0.50 [94] (0.27–0.92)	0.98 [140] (0.46–2.06)	0.55 [54] (0.37–0.82)	1.21 [44] (0.90–1.64)
*T*_max_ curve 2 (h)	2 (0.5–2.07)	2 (0.5–3.02)	1.00 (0.5–2.07)	1.76 (0.75–3.02)
AUC_0–6 h_ curve 1 (ng h/mL)	0.88 [124] (0.42–1.85)	2.01 [136] (0.97–4.17)	1.37 [45] (0.99–1.90)	3.35 [55] (2.31–4.84)
AUC_6–24 h_ curve 2 (ng h/mL)	0.98 [90] (0.54–1.77)	2.04 [115] (1.06–3.94)	1.47 [38] (1.11–1.95)	3.46 [47] (2.51–4.78)

^a^All parameters are presented as geometric mean [coefficients of variation %] (95 % confidence intervals)

*AUC* area under the curve, *CL/F* oral clearance, *t*_*1/2*_ half-life, *C*_*max*_ peak plasma concentration, *T*_*max*_ time to reach *C*_max_

**Table 4:** Geometric mean ratios of THC and 11-OH-THC

Parameters	THC	11-OH-THC
AUC_0–24_ (ng h/mL)	2.40 (1.83–3.16)	2.25 (1.82–2.77)
CL/F (L/h)	0.83 (0.63–1.10)	0.89 (0.72–1.10)
*t*_1/2_ (h)	1.00 (0.72–1.39)	0.88 (0.58–1.34)

Geometric mean ratio 1.5 versus 0.75 mg over one dosing interval (90 % CI)

**Fig. 2:**
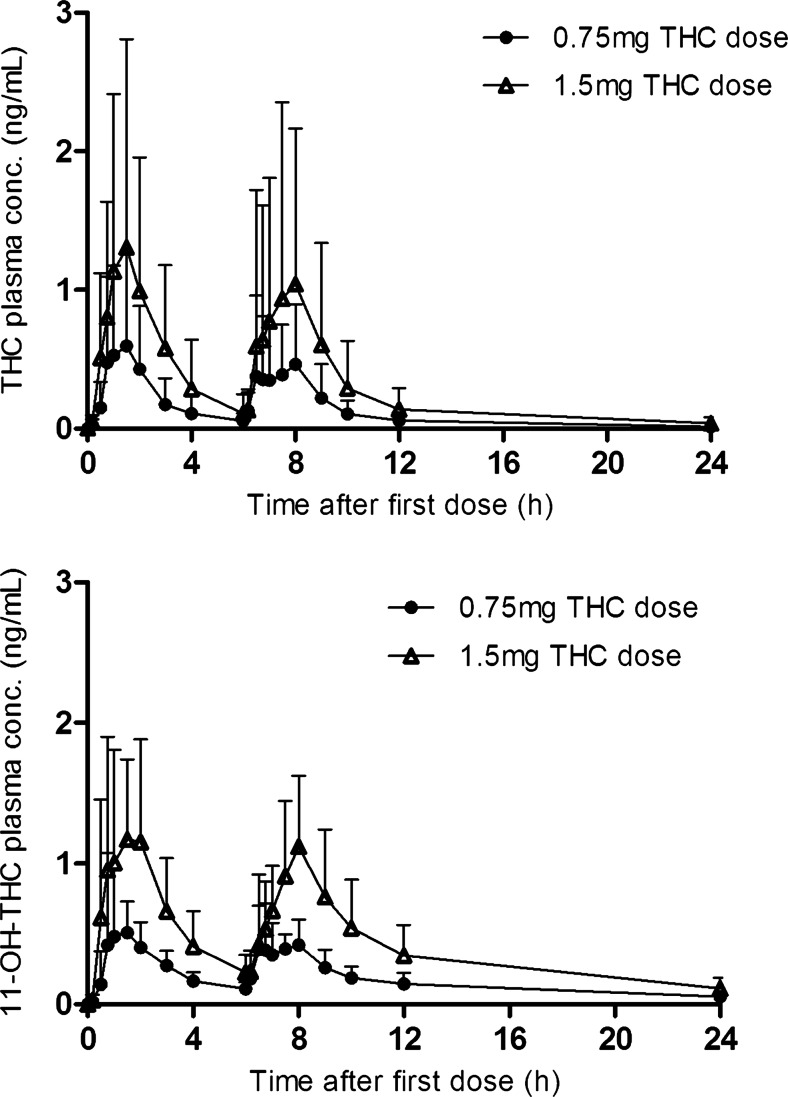
The mean concentration time profiles of THC and 11-OH-THC for both the 0.75- and 1.5-mg doses over 24 h

The median *T*_max_ was between 1 and 2 h and was not dose dependent. For the 0.75-mg dose, the median *T*_max_ was reached 1.5 h (range 0.75–3.08) after the first dose and 2 h (range 0.5–2.07) after the second dose; for the 1.5-mg dose, the median *T*_max_ was reached 1 h (range 0.5–2.2) after the first dose and 2 h (range 0.5–3.02) after the second dose (Table 3). Plasma concentrations of THC and 11-OH-THC increased linearly with increasing dose, but there was considerable interindividual variation in plasma concentrations and hence in pharmacokinetic parameters (Fig. 2). For THC, *C*_max_ and AUC CV% ranged from 90 to 140 %, and for 11-OH-THC from 38 % to 62 %. The elimination phase of THC was faster than that of 11-OH-THC.

**Fig. 3:**
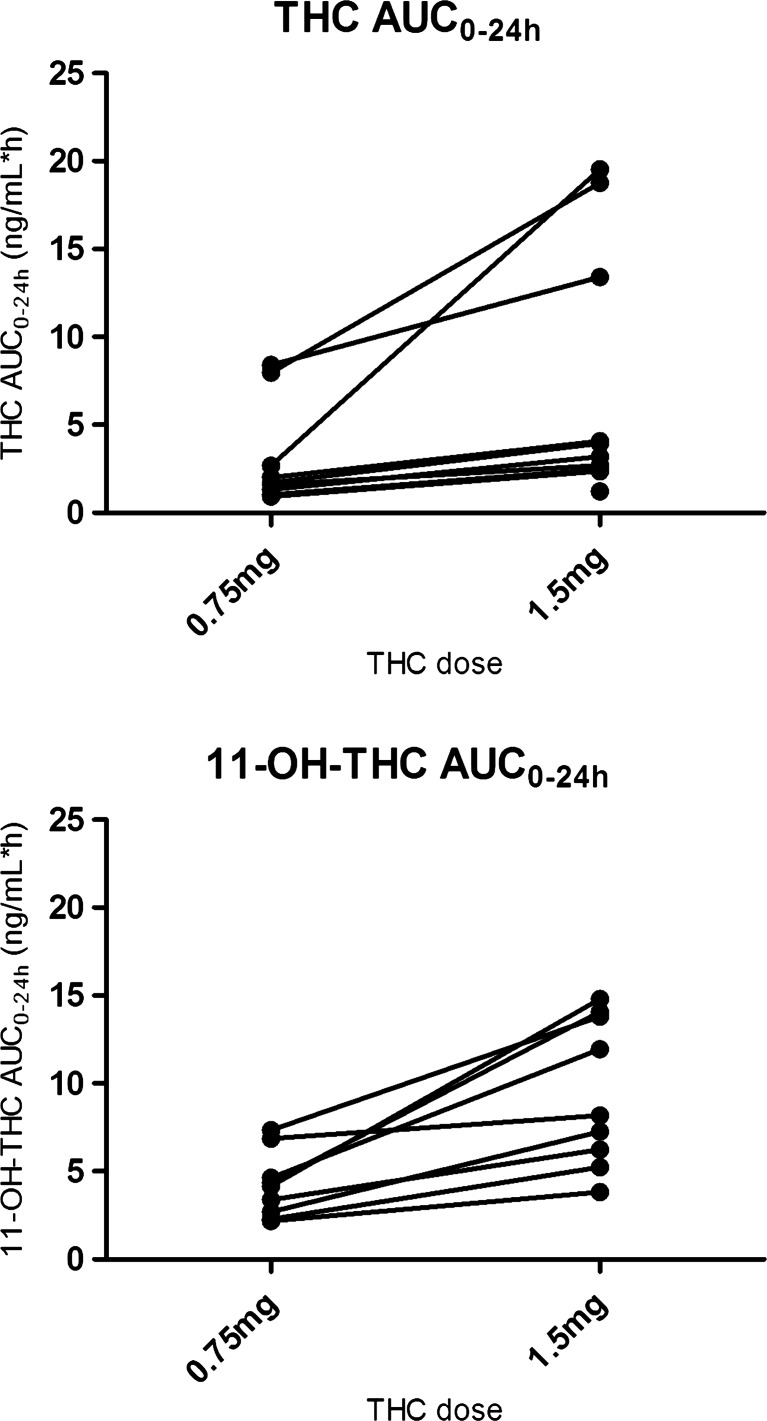
Individual pharmacokinetic parameter graphs

The geometric mean ratio of the THC AUC_0–24 h_ versus the 11-OH-THC AUC_0–24 h_ was 1.7 (95 % CI 1.1, 2.9) and 1.9 (95 % CI 1.0, 3.6) for the 0.75- and 1.5-mg doses, respectively. Individual THC and 11-OH-THC AUCs are presented in Fig. 3. Two participants had a high THC exposure after the 0.75-mg dose. Their AUC_0–24 h_ was 8.0 and 8.4 ng h/mL compared with a value ranging between 0.9 and 2.7 ng h/mL in the other participants. Three participants had a high exposure after the 1.5-mg dose. Their AUC_0–24 h_ was 13, 19, and 20 ng h/mL compared with a value ranging between 1.2 and 4.1 ng h/mL in the other participants. One participant had a greater increase in THC AUC after the 1.5-mg dose than the other participants; the AUC GMR for this subject was 7 compared with 1.7–2.5 (range) for the other participants. The same was seen for 11-OH-THC, but less pronounced (Fig. 3).

## Discussion

### Safety and tolerability

Older people with dementia and physical comorbidity could greatly benefit from the therapeutic application of cannabinoids. Recent studies have demonstrated that low doses of THC are effective in protecting the brain from neuroinflammation-induced cognitive damage (Aso et al. 2012; Aso and Ferrer 2014; Fishbein-Kaminietsky et al. 2014). Although THC-based drugs have recently been approved for clinical use, there are only few data on their safety in older individuals with dementia. Our data demonstrate that THC doses of 0.75 and 1.5 mg twice daily are safe and well tolerated by older individuals with dementia. Only 6 of the 98 reported adverse events were related to THC treatment. All adverse events were mild and resolved spontaneously without any intervention. Our findings are in line with previously published studies showing that THC doses up to 5 mg/day are safe to use in older individuals with dementia (Volicer et al. 1997; Walther et al. 2006, 2011). It is important to note that the safety data presented in this study are based upon short-term use of THC in older subject with dementia. Further studies are warranted to evaluate the long-term use of THC in this population.

### Pharmacodynamics

Overall, THC had fewer pharmacodynamic effects, including adverse events, than we had expected for frail older individuals with dementia, based on the effects reported by Klumpers et al. (2012) in young adults (mean age 21 years). We found no statistically significant changes in participants’ feeling high, external perception, body sway with the eyes open, and diastolic blood pressure after THC. The changes in internal perception, body sway with eyes closed, systolic blood pressure, and heart rate after THC were not considered clinically relevant, as they were small and were not associated with adverse events. The current findings are consistent with our previous findings from a phase 1 study of Namisol® in healthy older individuals without dementia (*n* = 11, mean age 72 years) (Ahmed et al. 2014a).

### Pharmacokinetics

On the basis of the AUC and *C*_max_ values, THC has linear pharmacokinetics in elderly individuals with dementia, showing a doubling of the AUC and *C*_max_ with doubling dose from 0.75 to 1.50 mg. However, there was considerable interindividual variation in plasma concentrations of THC and 11-OH-THC, which is in line with our data from a phase 1 study involving healthy older individuals (Ahmed et al. 2014a), and with the results of studies involving individuals of different ages (Carroll et al. 2004; Joerger et al. 2012; Klumpers et al. 2012). The median *T*_max_ was reached 1–2 h after THC dosing, as has been previously reported for healthy older individuals without dementia (Ahmed et al. 2014a). In contrast, Klumpers et al. (2012) reported a shorter *T*_max_ between 39 and 56 min in young adults after Namisol® administration. The AUC_0–6 h_ for older persons with dementia was two times higher than would be expected on the basis of data for young adults administered Namisol® (individual concentrations were retrieved and AUC_0-6h_ was calculated) (Klumpers et al. 2012). A possible explanation for the discrepancies in *T*_max_ and AUC_0–6 h_ is that, in the current study, THC was taken in nonfasting state, whereas Klumpers et al. (2012) administered THC to fasting young adults. Stott et al. (2013), in their investigation of the effect of food on the absorption and bioavailability of cannabinoids, found that the *T*_max_ for THC was reached about 2–2.5 h later in the fed state than in the fasting state: the mean AUC and *C*_max_ for THC and 11-OH-THC were onefold and threefold higher, respectively, in the fed state than in the fasting state. Age-related factors, such as delayed gastric emptying time, decreased splanchnic blood flow, decreased gastrointestinal motility, and decreased absorption surface, could also affect the absorption and bioavailability of THC in older individuals. It was not possible to compare our data with data from other pharmacokinetic studies involving older individuals with dementia because we did not find any relevant studies that reported data separately for this group.

The relatively high THC exposure in two participants seems to have been due to a diminished metabolism of THC to 11-OH-THC, as in both participants the 11-OH-THC/THC ratio of the AUC_0–24 h_ was less than 1 for both doses, whereas it was almost 2 in the other participants. However, the sum of 11-OH-THC plus THC AUC_0–24 h_ was higher in these two participants than in the other participants, but this higher THC exposure was not associated with adverse events.

### Strengths and limitations

The main strengths of the current study were, first, its design. In this randomized, double-blind, placebo-controlled, repeated crossover study, study staff and participants were masked to assignment and participants served as their own control. This design strengthened the validity of the safety and pharmacodynamic data. Second, our study is the first to evaluate the pharmacokinetics and pharmacodynamics of THC in older individuals with dementia, a frail subgroup of older persons. Therefore, this study can be added to the limited literature available on this subject.

The most notable limitation is that we probably used a very low THC dose-escalation regimen, 0.75 to 1.5 mg, as only 6 of the 98 reported adverse events were related to THC treatment and the pharmacodynamic effects were in general smaller than we had expected for this subgroup of older persons. A future dose-escalation study is required to determine the maximum tolerable dosage. This will help to maximize effectiveness while keeping side effects acceptable.

## Conclusion

Our findings suggest that low THC doses are safe and well tolerated by frail older persons with dementia. Oral THC was rapidly absorbed, showing dose-linear pharmacokinetics with maximum plasma concentrations being reached between 1 and 2 h after dosing, although there was considerable interindividual variability. Overall, THC showed smaller pharmacodynamic effects in frail older individuals than expected on the basis of data for young healthy adults. These reassuring data warrant further pharmacodynamic and efficacy studies with higher THC doses in older patients with dementia.
